# Knowledge Mapping Analysis of Public Health Emergency Management Research Based on Web of Science

**DOI:** 10.3389/fpubh.2022.755201

**Published:** 2022-03-09

**Authors:** Li Yang, Xin Fang, Junqi Zhu

**Affiliations:** School of Economics and Management, Anhui University of Science and Technology, Huainan, China

**Keywords:** public health, emergency management, bibliometric analysis, CiteSpace, VOSviewer

## Abstract

At present, major public health emergencies frequently occur worldwide, and it is of great significance to analyze the research status and latest developments in this field to improve the ability of public health emergency management in various countries. This paper took 5,143 related studies from 2007 to 2020 from the Web of Science as research object and used CiteSpace, VOSviewer, and other software to perform co-word analysis, social network analysis, and cluster analysis. The results and conclusions were as follows: (1) the related research identified three periods: the exploration, growth, and outbreak period; (2) chronologically: the relevant research evolved from medical and health care for major diseases to emergency management and risk assessment of public health emergencies and then researched the novel coronavirus (COVID-19) pneumonia epidemic; (3) clustering analysis of high-frequency keywords, identifying three research hotspots: “disaster prevention and emergency medical services,” “outbreak and management of infectious diseases in Africa,” and “emergency management under the COVID-19 pneumonia epidemic.” Finally, this study combined the data and literature analysis to point out possible future research directions: from the research of the COVID-19 pneumonia epidemic to the research of general major public health emergencies, thinking and remodeling of the national public health emergency management system, and exploring the establishment of an efficient international emergency management cooperation mechanism.

## Introduction

Public health is the science and technology that prevents diseases, prolongs life, and promotes health through organized efforts and informed choices to benefit public and private institutions, large and small communities, and all individuals in society (1). Since the twenty first century, with the advancement of globalization, the frequency and complexity of public health emergencies have been increasing (2), from SARS, H1N1 influenza, dengue fever to Ebola, MERS, the Zika epidemic (ZIKV), and the recent COVID-19 epidemic, which continues to affect the world. It poses a serious threat to the security of countries worldwide and has had a tremendous impact on people's well-being and social stability. According to the United Nations Political Declaration on Universal Health Coverage adopted at the relevant high-level meeting of the United Nations General Assembly in 2019, global health is placed at the core of development. Public health issues are related to the sustainable development of the world (3). Emergency management does not only refer to the management of response activities such as preventive preparedness, monitoring, and early warning, disposal and rescue, recovery, and reconstruction before, during, and later stages of an emergency, and the whole life cycle of an emergency (4), but also to the system's ability to respond quickly to emergencies and perform tasks on time (5). Shoaf et al. emphasized the role of public health in disaster risk reduction (6). Singleton et al. systematically discussed the synergy between emergency management and public health, the COVID-19 epidemic that emerged at the end of 2019 and spread worldwide (7). Various secondary and derivative problems caused by it have also become a major global public health emergency caused by compound disaster risk, which has brought tremendous impact and challenges to the construction of emergency management systems and capacity improvement of governments (8–10).

In recent years, research on public health emergency management has rapidly developed. However, there is a lack of literature reviews in this field at home and abroad. Therefore, this paper conducts a scientific and quantitative analysis of the relevant literature on “public health emergency management,” referencing relevant scholars to grasp the development context and frontier in the field systematically. Given this study used CiteSpace, VOSviewer, Ucinet, and other software to visually excavate the relevant literature on the theme of “public health emergency management” in the core database of the Web of Science, comprehensively and systematically combed the research status of public health emergency management from 2007 to 2020, and excavated the development trend and hotspot information in this field.

## Data Sources and Research Methods

### Data Sources

In this study, the core collection database of the Web of Science was selected as the literature source for data retrieval to ensure the data's reliability and authority. English search formula: TS = (“emergency management” or “continuity management” or “crisis management” or “emergency administrator” or “response management” or “disruption management”) and (“public health” or “public hygiene”); Index = SSCI, SCI; Time span = 2007–2020; Language: English; Document type: article or review; Database update date: December 26, 2020; Retrieval time: December 26, 2020, a total of 5,173 English literatures were obtained, and 5,143 valid literatures were obtained by using WOS literature output function and CiteSpace de-duplication function.

### Research Methods

CiteSpace is a visual network analysis software based on citation analysis theory and Java environment, which is gradually developed under the background of scientometrics, data, and information visualization (11, 12), showing the complex relationship implied in the citation. VOSviewer is a bibliometric analysis software based on the principle of co-citation, which is used to draw a map of scientific knowledge in various knowledge fields (13). The data collected were analyzed visually using the WOS data analysis module in CiteSpace5.5.R2. They obtained relevant information such as the number of articles published year by year, the issuing institutions, the source countries, and the core authors, to evaluate the research status of public health emergency management objectively. Then, VOSviewer was used to generate knowledge maps and related data such as “keyword co-occurrence network” and “author co-citation network” and to analyze the research hotspots and evolution context in this field. Finally, the results of the bibliometric analysis are summarized, and future research directions are discussed.

## Visual Analysis of Knowledge Map

### Analysis of Time Characteristics of Posting Volume

**Figure 1:**
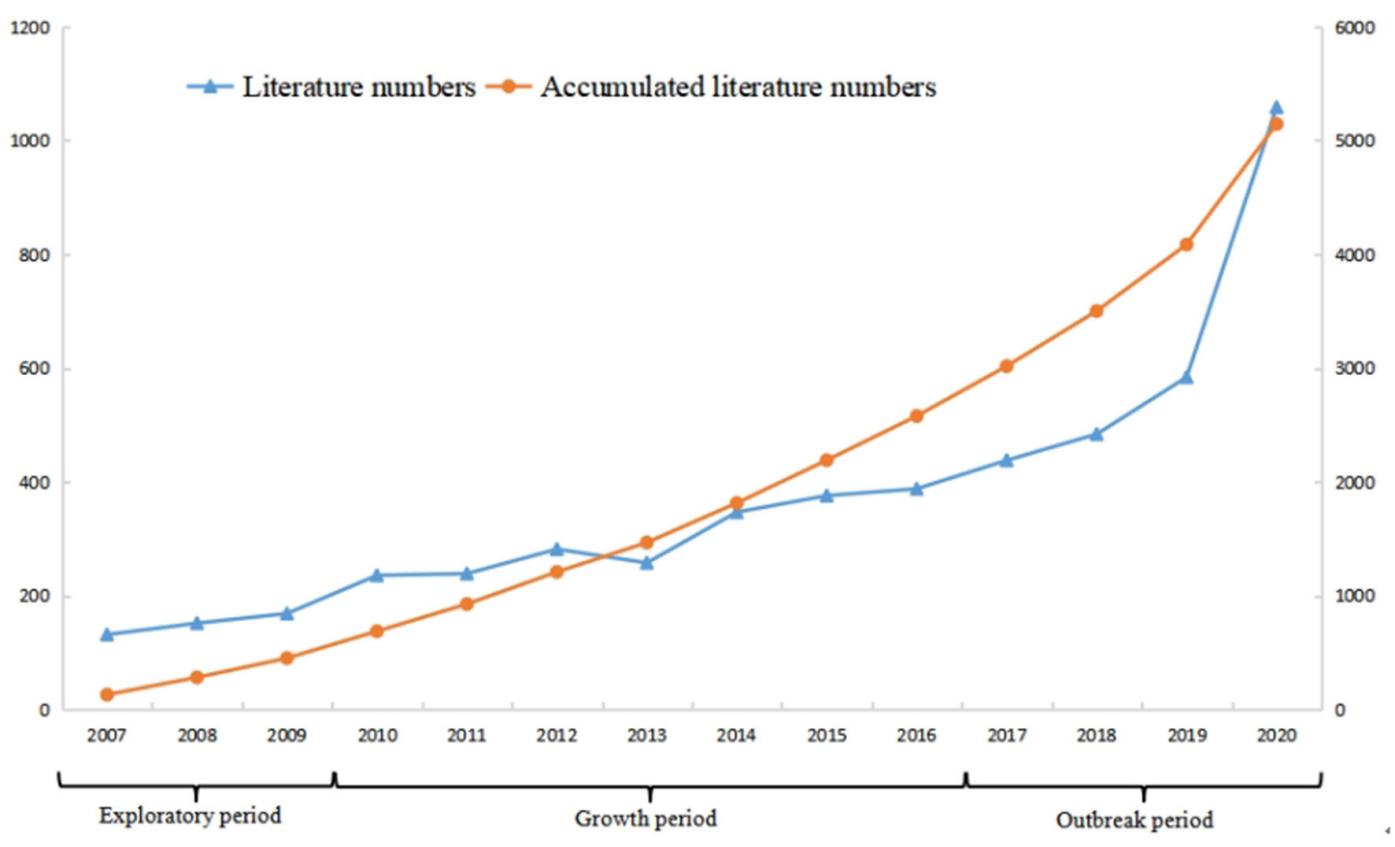
Annual publication of public health emergency management research.

The number of articles published on a specific topic in international academic journals represents the degree of concern of the research topic to a certain extent, and the data of annual publication volume and its growth rate can reflect the rise time of the research topic and the change in its degree of concern. Figure 1 shows that the earliest article was published in 2007. During the 3 years from 2007 to 2009, the number of papers issued increased year by year, with an average development rate of 113.15%, but the annual number of papers published was at a low level as a whole, which we defined as the initial exploration period of public health emergency management research. The number of papers published in 2010 was 236, with a growth rate of 39.64%, a peak in the curve of the number of papers published and regarded as a mutation value. During 2010–2016, the annual number of papers began to exceed 200, with an average development rate of 110.18%, and the cumulative number of studies was relatively high, which was defined as the continuous development period of the research. From 2017–2020, the average development speed of the number of papers published was 134.17%, showing a rapid explosive trend as a whole, and the number of published papers in 2020 was as high as 1,058, which was defined as the outbreak period. Affected by the COVID-19 pneumonia, public health emergency management research has been increasing annually, and the research fever has been continuously enhanced.

### Analysis of Published Journals

Understanding the journal distribution of citation literature can help researchers grasp research hotspots and frontiers in this field through appropriate journals. The distribution of journals with 35 or more papers in the WOS core collection is shown in Table 1. The top three journals with the highest number of papers were DISASTER MEDICINE AND PUBLIC HEALTH PREPAREDNESS (146 papers), PLOS ONE (125 papers), and BMC PUBLIC HEALTH (108 papers), accounting for 2.839%, 2.430%, and 2.100% of the sample, respectively. According to the overall distribution of published journals, there are seven journals with more than 40 papers, and these journals published 709 papers, accounting for 13.79% of the total. There are 14 journals with 20–39 papers, and the total number of journals is 1,716. This shows that journals related to public health emergency management research are relatively scattered. Among the seven journals with more than 40 papers, the impact factors of journals are all higher, and 50% of them are >2.4, which indicates that many authoritative journals in academic circles are interested in the research of public health emergency management.

**Table 1:** Distribution of literature source journals (top 10).

**Rank**	**Journal**	***N***	***P* (%)**	**IF**
1	DISASTER MEDICINE AND PUBLIC HEALTH PREPAREDNESS	146	2.839	0.977
2	PLOS ONE	125	2.430	2.740
3	BMC PUBLIC HEALTH	108	2.100	2.521
4	INTERNATIONAL JOURNAL OF ENVIRONMENTAL RESEARCH AND PUBLIC HEALTH	106	2.061	2.849
5	BMJ OPEN	101	1.964	2.496
6	BMC HEALTH SERVICES RESEARCH	78	1.517	1.987
7	PREHOSPITAL AND DISASTER MEDICINE	45	0.875	1.315
8	JOURNAL OF MEDICAL INTERNET RESEARCH	38	0.739	5.034
9	PUBLIC HEALTH	38	0.739	1.774
10	SCIENCE OF THE TOTAL ENVIRONMENT	35	0.681	6.551

### Analysis of the Cooperation Characteristics of the Issuing Country, Institutions, and Author

**Figure 2:**
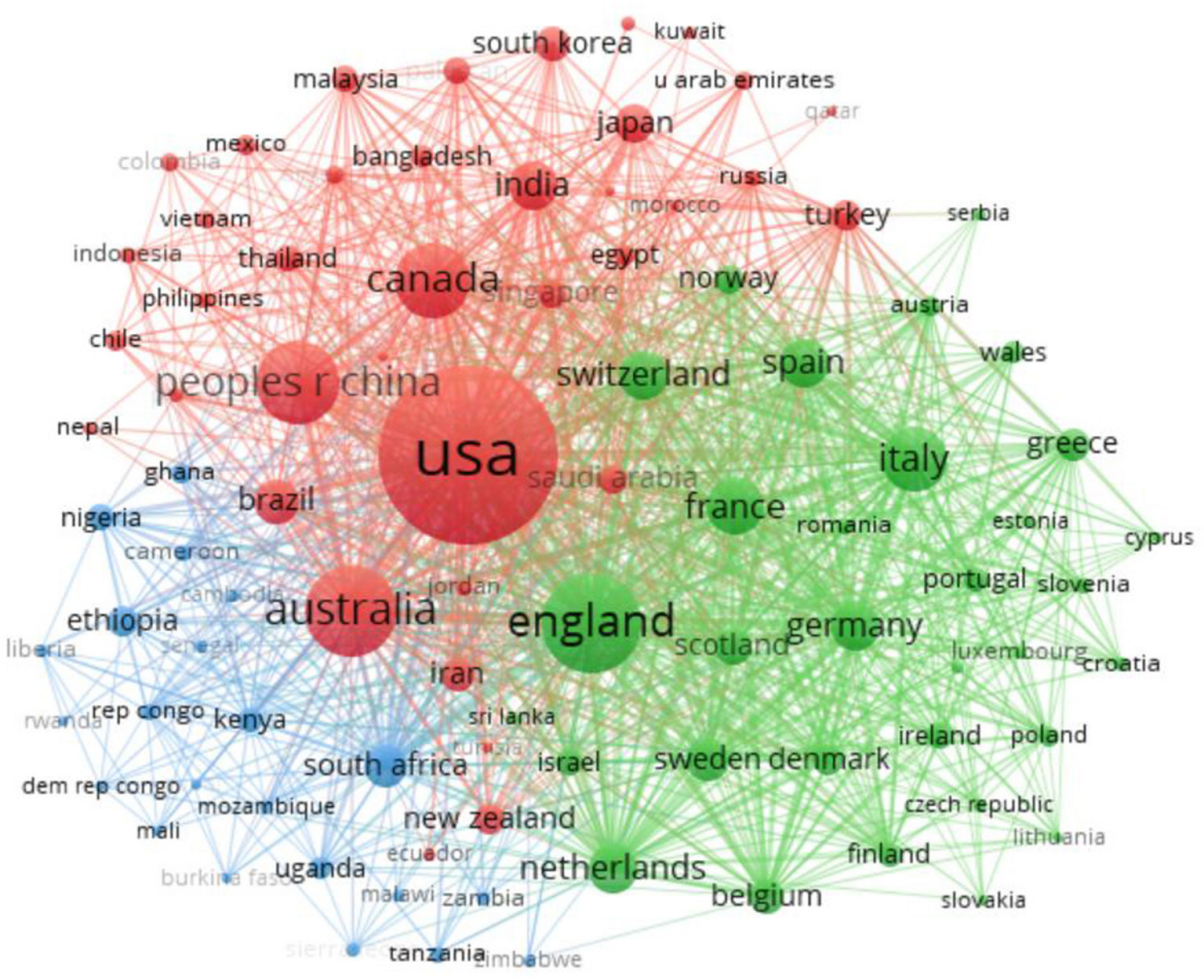
National atlas of research papers on public health emergency management from 2007 to 2020.

VOSviewer can draw national cooperation networks, institutional cooperation networks, and author cooperation networks and interpret the map to understand the different levels of cooperation in public health emergency management, to find the core countries, institutions, and individuals in the field. Figure 2 shows the map of cooperation among different countries, with 92 nodes and 1,725 links, which clearly shows that scholars in many countries attach great importance to public health emergency management research, and there is more cooperation among countries. In terms of the number of published papers, the United States obtains the most research results in the field of public health emergency management, with 2,120 papers published, accounting for 41% of the total number of published papers. In addition, its intermediary centrality is 0.02, which indicates that although the United States has a large number of papers, it lacks highly cited articles. There is more cooperation between the United States and China, while European countries take the United Kingdom as the center, and South Africa is relatively important in African countries, the cluster cooperation formed among many countries is close, and the network system is strong. In addition, the UK, Australia, China, Canada, and other countries have also published many high-impact papers in this field. The number of published papers is proliferating, and the intermediary centrality is constantly improving, indicating that an increasing number of countries have begun to pay attention to research in the field of public health emergency management.

Table 2 shows the top 10 research institutions in terms of intermediary centrality and frequency, including seven in the United States, one in the United Kingdom, and one in Australia. The institutions with the highest intermediary centrality and frequency are all from the United States, and among the top 10 institutions, the United States monopolizes seven. The United States has been in the leading position in this field based on its inception and duration of research. The top five institutions are Harvard University, the Centers for Disease Control and Prevention, the University of Sydney, the University of British Columbia, and the University of California, Los Angeles (UCLA). These institutions have solid academic strengths. Harvard University has world-class academic influence in the life sciences, natural sciences, and sociology. The United States Centers for Disease Control and Prevention (CDC) is the first federal health organization founded in the United States, focusing on the development and application of disease prevention and control, environmental health and other activities to improve people's health. The University of Sydney ranks 20th in the world in life medicine. The University of British Columbia enjoys a high reputation in management and hygiene, and UCLA is a world leader in medicine and business management.

**Table 2:** Top 10 institutions of literature output from 2007 to 2020.

**Number**	**Institution**	**Between centrality**	**Frequency**
1	Harvard Univ	0.13	106
2	Ctr Dis Control & Prevent	0.12	123
3	Univ Sydney	0.09	87
4	Univ British Columbia	0.09	65
5	Univ Calif Los Angeles	0.09	42
6	Univ Calif Irvine	0.09	14
7	Univ Massachusetts	0.08	14
8	Univ Washington	0.07	92
9	WHO	0.07	78
10	Columbia Univ	0.07	65

**Figure 3:**
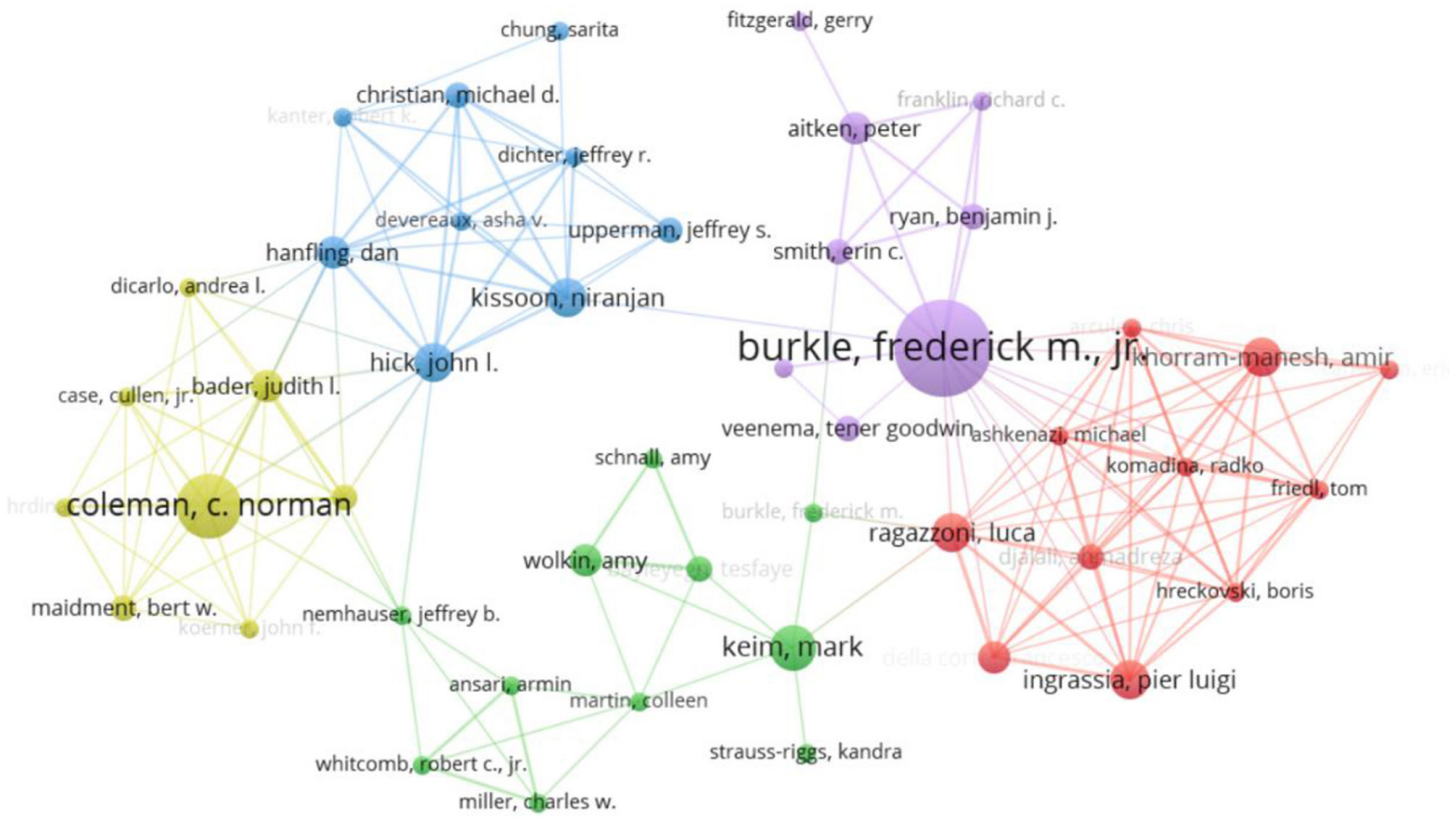
Cooperation network of authors in public health emergency management.

The development and evolution of disciplines benefit from the contribution of researchers, and researchers, as endogenous forces, can promote the development of disciplines. Through the authors' analyses, we can understand the general situation of the papers issued by the authors in this field and then realize the tracking and cooperative research in this field. Figure 3 shows the author collaboration network map in the field of public health emergency management; the top five authors in terms of published papers are Frederick M (10 papers), C Norman Coleman (6 papers), Carlos A (6 papers), Falko F Sniehorta (5 papers), and Amir Khorrammanesh (5 papers). The top five cited authors are Huang Cl (70 times), Moher D (65 times), Van Den B (63 times), Zhou F (52 times), and Liu Y (49 times). Each author is represented by a dot in the graph, and the collaboration between authors is represented by a line between nodes. From the map, we can see that several research cooperation teams have been formed, especially the team with Professor Amir Khorrammanesh as the core, which has carried out many cooperative research and established cooperative relations with many scholars. The overall level of cooperation among authors is low, and most researchers are individuals with little cross-team communication.

## Analysis on Hotspots of Public Health Emergency Management

### Literature Co-citation Analysis and Knowledge Base Identification

**Figure 4:**
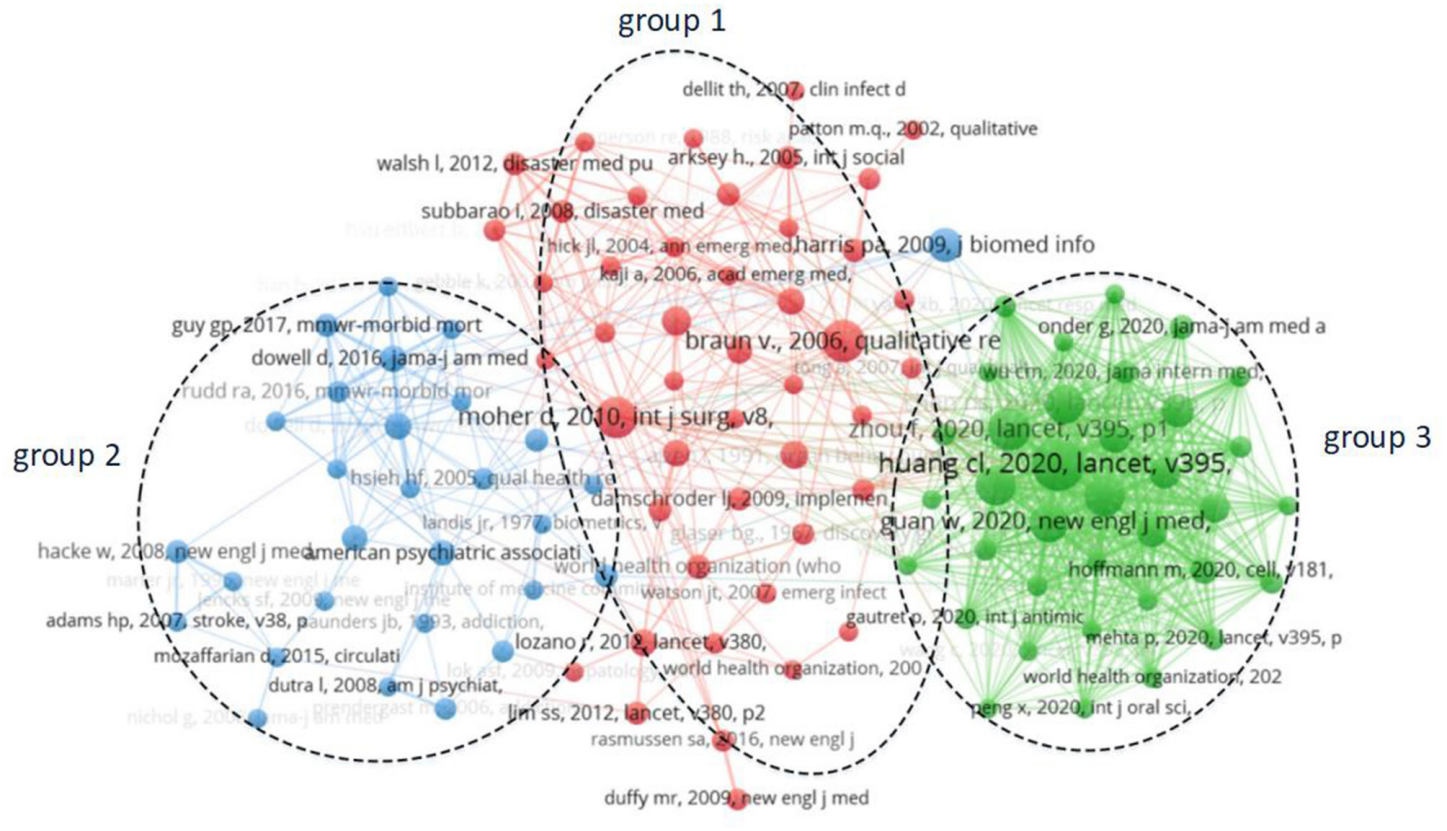
Visualization atlas of literature co-citation network.

Literature co-citation analysis is a research method to measure the degree of relationship between literature, exploring the development and evolution. There were 218,552 citations in 5,143 articles on public health emergency management collected in this study; the minimum number of citations was set as 12, and 112 citations were finally obtained. For the 112 selected studies, the modular layout and clustering method of VOSviewer were used to construct a visual map of the literature co-citation network, as shown in Figure 4.

In Figure 4, 112 nodes represent 112 studies, and the distance between nodes represents the similarity of the literature. The visualization results showed that 112 citations were automatically divided into three clusters according to the VOSviewer clustering method. The knowledge base of public health emergency management was divided into three knowledge groups by merging and summarizing the topics of every cluster: global disease and health services (knowledge group 1), guidelines for opioid use (knowledge group 2), and clinical characteristics of patients with the COVID-19 pneumonia (knowledge group 3).

1. Knowledge base 1: global disease and health services
  Knowledge group 1 focused on global diseases and health services. In 2009, Duffy et al. found for the first time that ZIKV spread outside Africa and Asia (14, 15), which helped clinicians and public health officials to realize the risk of further expansion. In 2014, Aylward et al. introduced forward-looking thinking to predict the Ebola virus (16) and proposed increasing control measures, which effectively reduced the virus's mortality. Emerging infectious diseases are a heavy burden on the global economy and public health, and scientific monitoring is needed (17). Walsh pointed out that the core competence of disaster medicine and public health is to develop a set of clear and concise training standards for health professionals to respond to major public health emergencies effectively (18). Damschroder proposed to put the research results of health services into practice and promote the implementation of a comprehensive scientific framework (19). In 2009, Harris combined electronic data capture with public health services for the first time (20), aiming to rapidly develop and deploy electronic data capturing tools to facilitate clinical and translational research.
2. Knowledge base 2: guidelines for opioid use
  Knowledge group 2 focused on guidelines for opioid use. Adams proposed early management guidelines for patients with acute ischemic stroke and pointed out that further research on the treatment of acute ischemic stroke is needed (21). Hacker tested the alteplase treatment in stroke patients, and the results showed that the treatment was more often associated with symptomatic intracranial hemorrhage (22). Brummett found that continuous opioid use was common after minor and major surgical procedures (23) and pointed out that new continuous opioid use will bring a previously underestimated surgical complication, worthy of further understanding. To effectively evaluate the effectiveness and risk of long-term use of opioids for the treatment of chronic pain, Chou found that the effectiveness of long-term opioid treatment to improve chronic pain could not be determined through randomized trials and observations (24), and excessive opioid treatment would lead to a risk of dependence. The United States Centers for Disease Control and Prevention has updated opioid prescription guidelines for chronic pain, to improve the safety and effectiveness of pain treatment and reduce the risks associated with long-term opioid treatment (25, 26).
3. Knowledge base 3: clinical characteristics of patients with COVID-19 pneumonia
  Knowledge group 3 focused on the clinical characteristics of patients with COVID-19 pneumonia. In 2020, Huang et al. collected and analyzed the data of patients infected with COVID-19 (27) and found that most of the infected patients were male. All patients had pneumonia, abnormal chest computed tomography, and complications, which revealed an urgent need to fill a major gap in the understanding of epidemiology in further studies. Guan found that the median age of COVID-19 patients was 47 years, and the most common symptoms were fever and cough, and the vast majority of patients developed lymphoma (28). Zhou described the risk factors leading to death and the detailed clinical disease course and that the long-term shedding of the virus justifies future isolation of infected patients and optimal antiviral interventions (29). Hoffmann proposed insights into which cytokines in COVID-19 might provide viral transmission and identified potential targets for antiviral intervention (30). In 2020, Wu, for the first time, systematically summarized the major epidemiology and clinical findings of all COVID-19 cases reported in the Chinese mainland (31). He also reported case trends in response to the government's attempts to control the infection, emphasizing that aggressive investment in public health infrastructure is essential for an effective response to outbreaks, the continued improvement of international surveillance, cooperation, coordination, and communication on this pandemic, which will contribute to a better response to new threats to public health.

### Keywords Frequency Statistics and Co-occurrence Matrix

Keywords can well reflect the main content of the paper and are highly generalized and concise of the research topic. High-frequency keywords usually reflect current issues and frontier trends in a specific research field, so the statistical analysis of literature keywords can quickly and effectively understand the research hotspots in this field. Bibexcel was used to analyze the keywords of the published literature through keyword frequency analysis technology, and keywords with similar meanings were merged, keywords unrelated to this study were eliminated, and keywords with frequencies ≥15 were selected as high-frequency keywords for further analysis. Table 3 lists the top 20 keywords.

**Table 3:** Common word matrix of high-frequency keywords (part).

**Rank**	**Keywords**	**Frequency**	**Rank**	**Keywords**	**Frequency**
1	Public health	347	9	Knowledge	44
2	COVID-19	347	10	Prevention	43
3	Epidemiology	168	11	Risk assessment	43
4	Disaster	62	12	Emergency management	43
5	Emergency department	54	13	Climate change	42
6	Health policy	54	14	Education	40
7	Primary care	51	15	Emergency preparedness	40
8	Management	50	16	Mental health	39

### Keyword Co-occurrence Analysis and Research Hotspot Identification

**Figure 5:**
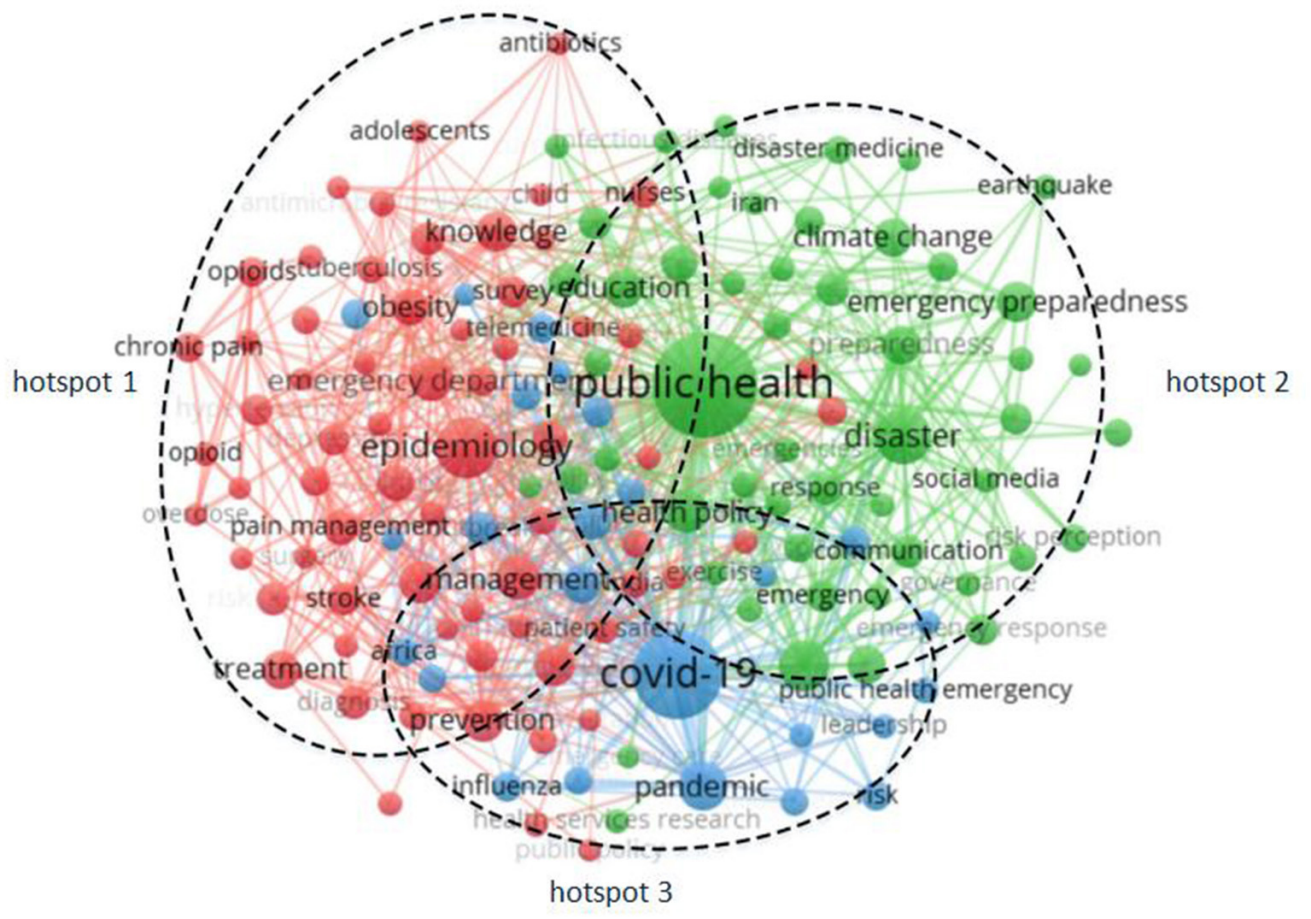
Keywords co-occurrence network visualization map.

**Figure 6:**
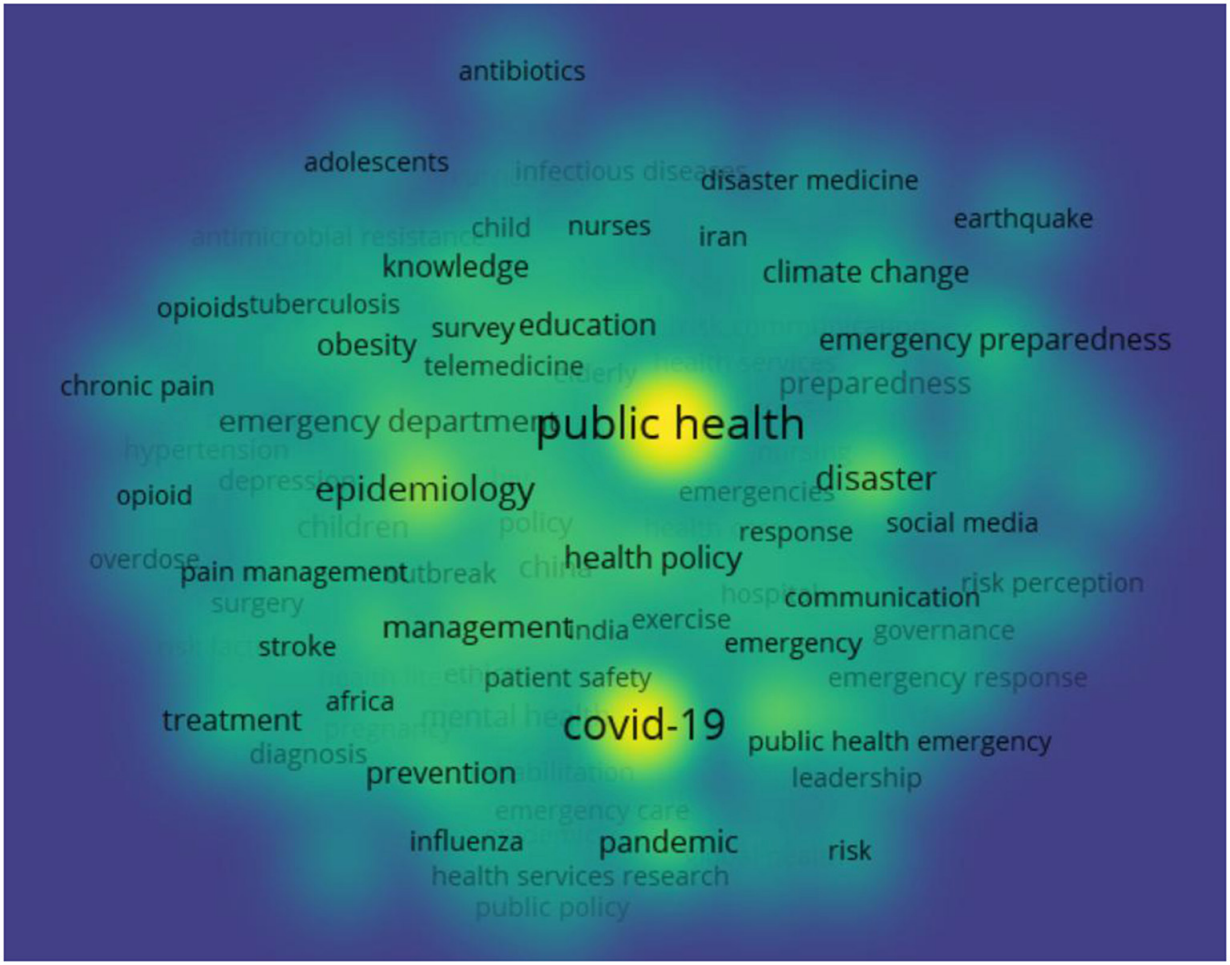
Keywords co-occurrence clustering density visualization map.

Keyword co-occurrence analysis is a method that uses statistical methods to calculate the frequency of co-occurrence of keywords in the same document, obtains a co-occurrence matrix, and then converts the co-occurrence matrix into a co-occurrence network (32). This study systematically combs all the keywords of 5,143 articles. It generates the co-word matrix by Bibexcel, utilizing the modular clustering algorithm of VOSviewer to analyze the co-occurrence of 129 keywords and obtain the visual map of the keyword co-occurrence network and the visual map of the keyword co-occurrence cluster, as shown in Figures 5, 6.

It can be seen from the visualization results in Figure 5 that the co-occurrence network of 129 keywords forms three clusters. Three research hotspots in the field of public health emergency management are obtained by summarizing the literature topics of each cluster in combination with citation analysis, namely, disaster prevention and emergency medical services (hotspot 1), including public health, emergency preparedness, disaster planning, health policy, and emergency medical services. Hotspot 2 includes the outbreak and management of infectious diseases in Africa, including epidemiology in Africa, management, treatment, etc. Emergency management under the COVID-19 pneumonia epidemic (hotspot 3), including keywords like COVID-19, China, attitude, management, and more. It can be seen from Figure 6 that public health, COVID-19 pneumonia, and epidemiology are becoming the research focus of public health emergency management in the yellow area, and other research can be regarded as being carried out around these three cores.

1. Research focus 1: disaster prevention and emergency medical services
  Hotspot 1 focused on disaster prevention and emergency medical services. Abir et al. suggested that professional associations could use their member networks to collect survey data promptly to inform best practices during and after public health emergencies (33). In 2018, Acosta et al. argued that the network of community-based organizations has a flexible range of partner services and that coordinating community and public health partnerships can effectively contribute to disaster recovery (34), which provides a new way to deal with major public health emergencies. Adini et al. suggested that familiarity with guidelines and preparedness assessments affects healthcare managers' perceptions of their ability to prepare for and manage pandemic influenza (35). Abdullelah et al. believed that strengthening nurses' knowledge of core competency areas can contribute to disaster recovery and proposed the involvement of stakeholders in the adaptation process in response to the Australian heatwave disaster (36). In 2014, Aung et al. proposed the traceability of food supply chains for the first time to effectively ensure food quality and safety, aiming to help consumers achieve confidence (37). In 2010, Huang et al. proposed for the first time that Web2.0 and Internet social network are new tools for disaster management and established an Internet-based emergency response system to prevent disasters (38), which put forward new measures for public health emergency management from the perspective of big data networks.
2. Research hotspot 2: outbreak and management of infectious diseases in Africa
  Hotspot 2 focused on the outbreak and management of infectious diseases in Africa. Africa's economy is backward, healthcare is extremely scarce, and how to deal with the spread of infectious diseases is gradually becoming the focus of many scholars. Chopra et al. argued that South Africa needs talented leadership, vision, and commitment to meet new challenges and address public health emergencies (39). In 2019, Bedford et al. put forward a new concept of the epidemic (40), which evolved from a crisis response of discrete outbreaks to an integrated cycle of preparedness, response, and recovery, which required improving countries' public health emergency management capacity. Critchley et al. suggested that due to the convergence of epidemics in sub-Saharan Africa, research should prioritize addressing the interaction among TB, diabetes, and HIV (41). Migliori et al. argued that with the current global trend of MDR-TB increasing, existing interventions, public health systems, and TB programs must be significantly strengthened, and political and funding commitments are essential to curb the spread of MDR-TB (42). Peyre et al. studied the different economic impacts caused by Rift Valley Fever and proposed that early detection and rapid response should be implemented to help decision-makers make choices related to its management (43).
3. The research hotspot 3: emergency management under COVID-19 pneumonia epidemic
  Hotspot 3 focused on emergency management during the COVID-19 pneumonia epidemic. The spread of COVID-19 globally has caused huge casualties and property losses, destroyed public health order, and caused great social panic. Based on this background, many countries and institutions have conducted in-depth research. Al-Shamsi et al. pointed out that many cancer patients are at higher risk of infection due to low immune function and proposed telemedicine to help patients reduce the number of visits and exposure risks (44). In 2020, Chan et al. argued that in addition to a top-down health emergency and disaster risk management, bottom-up individual and household measures are essential for COVID-19 prevention and emergency response (45), which is conducive to raising public awareness of self-protection. In the face of the new epidemic, governments have taken a series of rapid, comprehensive, and effective prevention and control measures, established and improved the public health emergency response system, especially the emergency reserve system of medical supplies, and promoted the establishment of international cooperation projects to respond to international public health emergencies jointly (46–48). In 2020, Zhang et al. believed that risk communication was essential to public health emergency management and constructed a simplified government-expert-public risk communication model to illustrate the important role of an effective risk communication collaboration network in dealing with the COVID-19 epidemic (49), which provided new countermeasures and suggestions for public health emergency management.

### Analysis on the Evolution Trend of Public Health Emergency Management

**Figure 7:**
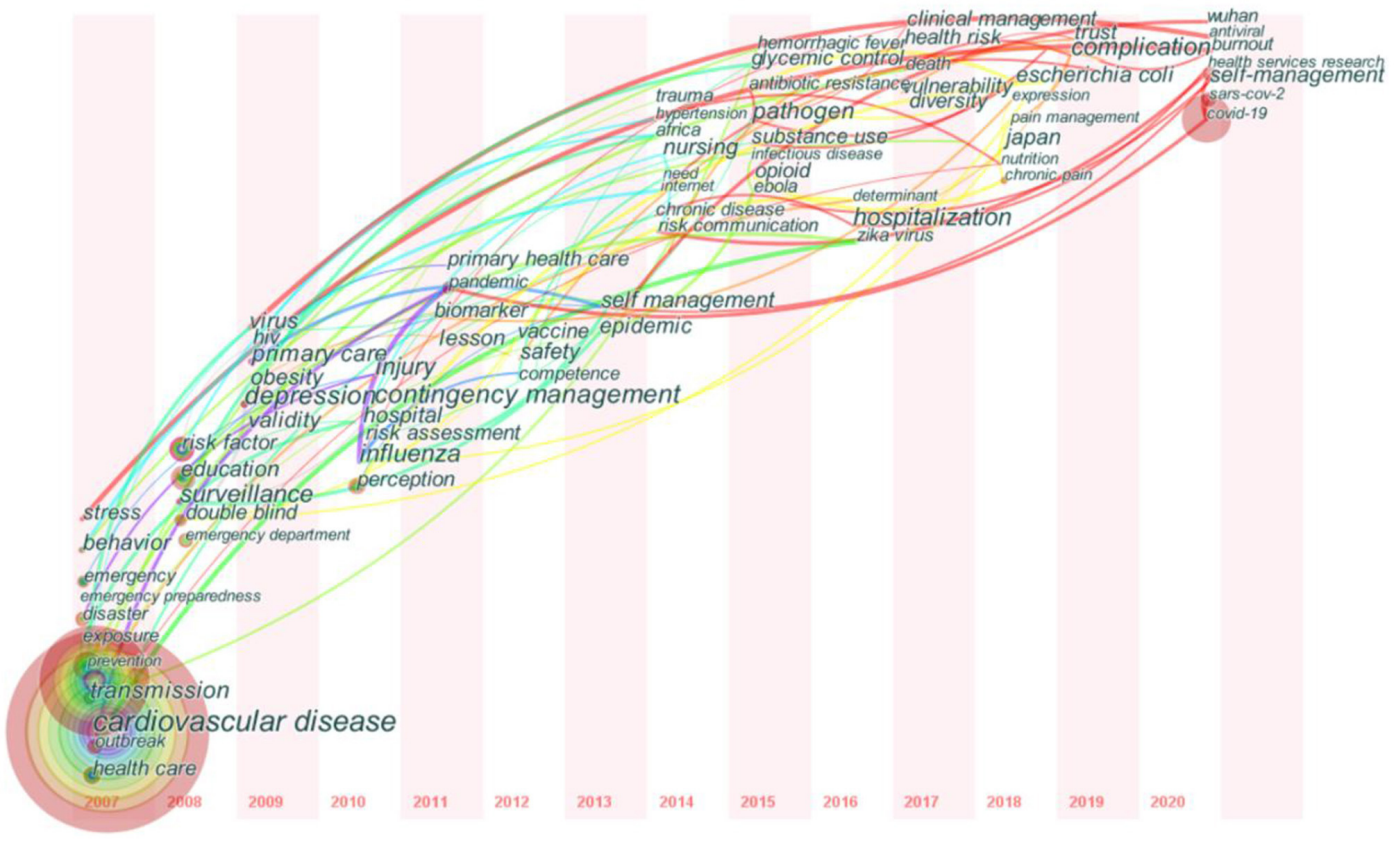
Keywords time zone evolution map.

To better understand the development trend in the field of public health emergency management in recent years, this paper presents a time zone map of keywords in public health emergency management research based on the analysis map of keyword co-occurrence cooperation network (Figure 7). The time division was set as 1 year, the node type was set as a keyword, and the knowledge map was drawn using the pathfinder algorithm, which resulted in 509 keywords and 720 node connections. The size of the nodes in the graph indicates the frequency of keywords, and the connections indicate the co-word relationships between keywords. With the help of the update and interaction of articles presented by the keyword time zone map, the evolution process of public health emergency management research from 2007 to 2020 is revealed, and the migration path of research hotspots can be clearly understood through the time zone map.

Public health emergency management initially focused on health research, such as cardiovascular disease (2007–2009). Cardiovascular disease refers to a series of diseases caused by heart and vascular diseases, including coronary heart disease, hypertension, and cerebrovascular disease, among others, with the characteristics of high morbidity, high disability rate, high recurrence rate, high mortality rate, and many complications, coupled with the limited medical and health technology at that time, according to the World Health Organization. Among the major diseases threatening human health, cardiovascular disease has the highest mortality rate, which has become a major global health problem and poses serious challenges to the world. This quickly became a research hotspot at that time.

With the deepening of research, the focus of research in this field has shifted to emergency management and risk assessment of public health emergencies (2010–2018). Since the twenty first century, the frequency of major public health emergencies and their derivative crises has been increasing, and there is an urgent need to do a good job in public health emergency management and risk assessment. Pre-disaster prevention and early warning mechanisms have been put in the core position, and the comprehensive assessment of public health risks improves the ability to deal with major public health emergencies.

In recent years, public health emergency management has focused on the COVID-19 epidemic (2019–2020). The COVID-19 epidemic is the most serious public health crisis in human history, which has a fundamental impact on the global healthcare system and the national emergency management system, causing huge casualties and property losses. Many scholars at home and abroad have carried out in-depth research on the background, evolution path, response measures, and future development direction. It is emphasized that the major epidemic prevention and control system and mechanism and the national public health emergency management system should be constantly improved. International cooperation should be strengthened to combat the COVID-19 epidemic and other major public health emergencies, and public health emergency management is increasingly showing a trend of diversification.

## Discussion

In the past 5 years, the relevant literature on public health emergency management has been increasing. The life cycle theory shows that an event will go through gestation to extinction, and each stage has different characteristics (50). Accordingly, public health emergency management research will also experience a development process from less to more, from rising to falling. This study explores the life cycle of public health emergency management. According to the literature and development trends, three periods were identified: the initial exploration period, the continuous development period, and the outbreak. Although it is impossible to accurately predict the future life cycle of public health emergency management, this research remains an important topic, and it has a broad application prospect and theoretical value from the development trends.

In recent years, with the advancement of globalization, the frequency and complexity of public health emergencies have been increasing. Various secondary and derivative problems caused by the COVID-19 epidemic have also become a major global public health emergency caused by compound disaster risk, which has brought tremendous impact and challenges to the construction of emergency management systems and capacity improvement of governments. Emergency management does not only refer to the management of response activities such as preventive preparedness, monitoring, and early warning, disposal and rescue, recovery, and reconstruction before, during, and later stages of an emergency, and the whole life cycle of an emergency, but also to the system's ability to respond quickly to emergencies and perform tasks on time. Good emergency management can effectively deal with public health emergencies, which will help countries to establish and improve public health emergency management system and international emergency management cooperation mechanism, and improve the international community's ability to respond to major public health emergencies.

In the initial exploration period, theoretical research on this subject was in its early stage, and the average annual number of articles was <200. During this time, relevant research mainly focused on basic medical health research, such as stroke (51), influenza (52), cardiovascular disease (53), and so on. However, during this stage, the research field had certain limitations. Specifically, the high mortality rate of these diseases was a huge obstacle to be overcome in the medical field. In addition, the medical health level was low at that time, researchers focused more on public health and studied how to overcome these diseases. The relevant research on public health emergency management was relatively shallow.

In the continuous development period, the average annual number of articles was more than 200, and researches began to focus on emergency management and risk assessment of public health emergencies (54). With the excessive pursuit of high-speed economic growth, human beings have brought a serious burden to nature, the ecological environment has been seriously damaged, the frequency of major public health emergencies and their derivative crises are increasing, coupled with the impact of traditional public health events, scholars began to pay attention to emergency management and risk assessment. Kebede et al. believed that we should focus more on developing hospital managers, not just on enhancing medical and public health skills, recognizing the importance of management capacity to achieve sustainable development (55). Geographic information system can effectively reveal the differences of regional medical services, especially for primary health care and clinical health (56), and the use of health information exchange network can effectively evaluate regions medical health level (57). With the further implementation of telemedicine technology, the spatial distance is greatly reduced, which brings great convenience to the emergency management of public health emergencies (58). For low- and middle-income countries, we can assess current and planned pharmacovigilance activities, identify gaps and the most urgent pharmacovigilance priorities at national and international levels, and define the elements of a sustainable global pharmacovigilance strategy (59). Through pre disaster prevention, establishing and improving the early warning mechanism and further relying on science and technology, we can effectively improve the global ability to respond to major public health emergencies.

In the outbreak period, public health emergency management has focused on the COVID-19 epidemic. The COVID-19 epidemic, which appeared in 2019, has been spreading rapidly, with high uncertainty and high mortality rate. It has spread to most countries in the world, and has brought huge casualties and property losses, which posed severe challenges to global public health, and many researchers have turned to the COVID-19 epidemic research. Deshmukh et al. systematically summarized the updated epidemiology, causes, clinical manifestation and diagnosis, as well as prevention and control of the novel coronavirus SARS-CoV-2, which is essential to both manage the current pandemic and to conceive comprehensive measures to prevent such outbreaks in the future (60). Communities are central to the practice of public health emergency preparedness and response (61), it is important to identify the source of infection at the community level as soon as possible to block the transmission path of the virus to prevent the spread of the pandemic. The implementation of grid management in the community and the adoption of precise management and control measures to reduce unnecessary personnel movement can effectively reduce the risk of pandemic spread, and improving community disaster resilience are an effective method for coping with such a public health emergency (62). By analyzing the consequences of exclusionary othering in public health, Tallarek et al. proposed to move toward inclusionary and diversity-sensitive public health (63). Public health emergencies will lead to public health crisis, government risk communication plays a key role in responding to public health emergencies (64). Governments should timely disseminate and update information related to the epidemic, stabilize public sentiment, strengthen public opinion and epidemic prevention and control, and implement hierarchical management in key epidemic areas and urban agglomerations. In the face of public health emergencies, there will be deficiencies in biosafety, food safety, public health investment and emergency system construction (46), the world should constantly improve the system of emergency reserve medical supplies, promote the establishment of international cooperative programs to jointly deal with public health emergencies of international concern in the future. Public health emergency management is increasingly showing a trend of diversification.

Finally, this study combined the data and literature analysis to point out possible future research directions: from the research of the COVID-19 pneumonia epidemic to the research of general major public health emergencies, thinking and remodeling of the national public health emergency management system, and exploring the establishment of an efficient international emergency management cooperation mechanism.

## Limitations

The main limitation of this study is its focus on the published literature in English. Consequently, relevant information in other languages may be missing. The criteria used to narrow the selection of included publications enabled the authors to access eligible data and a feasible number of publications to handle the content analysis and to perform the review. However, the criteria used may have been too selective, resulting in missing information. In addition, in the selection of database, this study selects the representative Web of Science as the literature collection database, missing other databases. These limitations can be further addressed as a part of the future research.

## Conclusions and Recommendations

In this study, CiteSpace and VOSviewer, two authoritative scientometrics analysis software, were used to visually analyze 5,143 articles on public health emergency management in the core database of Web of Science. Based on bibliometric analysis methods, such as frequency analysis of keywords, analysis of the occurrence of keywords, and analysis of the time zone of keywords, this article combines the research status and hotspots in the field of emergency management in public health. It points out the following research direction and corresponding proposals. First, the regional distribution of scientific research forces in public health emergency management is unbalanced. The United States, the United Kingdom, Australia, and China are the top four countries in public health emergency management, accounting for 73.7% of the total literature. Among them, the United States carried out research on public health emergency management earlier, published the largest number of papers in this field, and had a larger impact coefficient, which was in the core position in this field. China ranks fourth in terms of the number of papers issued and has developed rapidly in recent years, with a large fund investment. Although the public health emergency management originated in developed countries, research on developing countries such as China, South Africa, and India is gradually increasing. In addition, the degree of cooperation between research institutions and core authors in this field is low, the number of high-yielding authors is small, most researchers are individuals, and cross-team communication is minimal. Therefore, collaboration is necessary to deepen cooperation and cultivate more research centers with vast influence.

Second, public health emergency management has shifted from focusing on post-disaster recovery to emphasizing pre-disaster prevention. With the acceleration of globalization, many public health emergencies have brought great threats and losses to people worldwide. An increasing number of scholars have begun to pay attention to pre-disaster prevention, resolutely implement the health policy of prevention first, and establish awareness of periodic risk prevention and control. They are establishing an Internet-based emergency response system, training a large number of professional public health personnel, and strengthening the public health infrastructure, which is aimed at the rapid response to major public health emergencies and disaster prevention.

Third, to fully understand the current status of research on public health emergency management, this study selected software especially suitable for bibliometric analysis, including CiteSpace, vosviewer, Netdraw, etc., conducting a visualization analysis based on the relevant research obtained from the Web of Science database, and determining the number of research studies, the major countries publishing on this topic, and the journal distribution. Then, keyword co word analysis, social network analysis and cluster analysis are selected to clarify the development trend and research theme of public health emergency management research hotspots. Finally, by reading and summarizing a large number of relevant literature, we have a good grasp of public health emergency management, and put forward possible future research directions, which fills the gap in public health emergency management and provides a framework for future research. At the same time, there are some limitations in. In the selection of database, this study selects the representative Web of Science as the literature collection database, other databases may have been missed. These limitations can be further addressed as a part of the future research.

Fourth, the construction of major epidemic prevention and control systems, mechanisms, and national public health emergency management systems have attracted much attention. The COVID-19 epidemic sweeping the world has seriously impacted the healthcare systems and emergency management systems of countries worldwide, causing massive casualties and property losses. We should strengthen epidemic prevention and control, innovate major epidemic prevention and control measures in the system and mechanism, clarify the prominent importance of building a major epidemic prevention and control system and mechanism and national public health emergency management system, and give full play to the main role of the government, market, and society; establish and improve an efficient monitoring and early warning system and emergency support system; and improve emergency plans and other related systems, which will improve the world's ability to detect public health emergencies on time, make scientific decisions, and jointly prevent and control them.

Fifth, the international emergency management cooperation mechanism for major public health emergencies needs to be further improved. Major public health emergencies are sudden, complex, and unbounded, which will produce a global “ripple effect.” The COVID-19 epidemic has extremely exposed the fragility of the international emergency management cooperation mechanism. It is necessary to strengthen the consensus of the “community of human destiny,” to improve the ability of the international community to respond to major public health emergencies. Countries should not shirk their responsibilities, adhere to the guiding principles of multilateral cooperation to jointly combat the epidemic, and promote the establishment of cross-regional, cross-sectoral, and cross-sectoral emergency management procedures and safeguards to give full play to the inherent advantages of the international emergency management cooperation mechanism.

## Data Availability Statement

The raw data supporting the conclusions of this article will be made available by the authors, without undue reservation.

## Author Contributions

LY and XF contributed to the analysis and interpretation of data for the study and wrote the first draft of the manuscript. XF designed the framework for this study. JZ contributed to the acquisition of data for this study. All authors approved the final manuscript.

## Funding

This study was supported by General Project of the National Natural Science Foundation of China (grant no. 71971003) and the Major Project of the National Social Science Funding of China (grant no. 20ZDA084).

## Conflict of Interest

The authors declare that the research was conducted in the absence of any commercial or financial relationships that could be construed as a potential conflict of interest.

## Publisher's Note

All claims expressed in this article are solely those of the authors and do not necessarily represent those of their affiliated organizations, or those of the publisher, the editors and the reviewers. Any product that may be evaluated in this article, or claim that may be made by its manufacturer, is not guaranteed or endorsed by the publisher.
